# 2-(2-Methyl-5-nitro-1*H*-imidazol-1-yl)ethyl 4-fluoro­benzoate

**DOI:** 10.1107/S1600536812006319

**Published:** 2012-03-03

**Authors:** Sammer Yousuf, Aurang Zeb, Fatima Z. Basha

**Affiliations:** a HEJ Research Institute of Chemistry, International Center for Chemical and Biological Sciences, University of Karachi, Karachi 75270, Pakistan

## Abstract

In the title compound, C_13_H_12_FN_3_O_4_, the dihedral angle between the benzene and imidazole rings is 32.77 (12)°. In the crystal, mol­ecules are linked into a three-dimensional network by C—H⋯O hydrogen bonds.

## Related literature

For biological activities of metronidazole derivatives, see: Atia (2009 ▶); Beena *et al.* (2009 ▶); Bowden & Izadi (1998 ▶); Dubey *et al.* (2009 ▶); Mao *et al.* (2009 ▶); Qian *et al.* (2010 ▶).
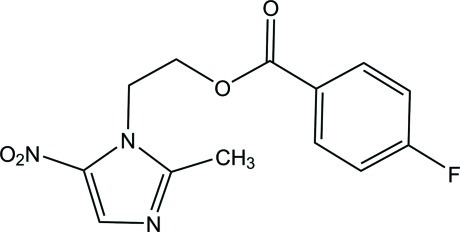


## Experimental

### 

#### Crystal data

C_13_H_12_FN_3_O_4_
*M* *_r_* = 293.26
Monoclinic, 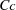

*a* = 8.9669 (12) Å
*b* = 18.784 (2) Å
*c* = 7.8288 (10) Å
β = 99.684 (3)°
*V* = 1299.9 (3) Å^3^
*Z* = 4
Mo *K*α radiation
μ = 0.12 mm^−1^
*T* = 273 K
0.50 × 0.29 × 0.16 mm

#### Data collection

Bruker SMART APEX CCD diffractometer
Absorption correction: multi-scan (*SADABS*; Bruker, 2000 ▶) *T*_min_ = 0.941, *T*_max_ = 0.981
3782 measured reflections
1186 independent reflections
1150 reflections with *I* > 2σ(*I*)
*R*_int_ = 0.022

#### Refinement

*R*[*F* ^2^ > 2σ(*F* ^2^)] = 0.032
*wR*(*F* ^2^) = 0.088
*S* = 1.06
1186 reflections
192 parameters
2 restraints
H-atom parameters constrained
Δρ_max_ = 0.15 e Å^−3^
Δρ_min_ = −0.11 e Å^−3^

### 

Data collection: *SMART* (Bruker, 2000 ▶); cell refinement: *SAINT* (Bruker, 2000 ▶); data reduction: *SAINT*; program(s) used to solve structure: *SHELXS97* (Sheldrick, 2008 ▶); program(s) used to refine structure: *SHELXL97* (Sheldrick, 2008 ▶); molecular graphics: *SHELXTL* (Sheldrick, 2008 ▶); software used to prepare material for publication: *SHELXTL*, *PARST* (Nardelli, 1995 ▶) and *PLATON* (Spek, 2009 ▶).

## Supplementary Material

Crystal structure: contains datablock(s) global, I. DOI: 10.1107/S1600536812006319/is5064sup1.cif

Structure factors: contains datablock(s) I. DOI: 10.1107/S1600536812006319/is5064Isup2.hkl

Supplementary material file. DOI: 10.1107/S1600536812006319/is5064Isup3.cml

Additional supplementary materials:  crystallographic information; 3D view; checkCIF report

## Figures and Tables

**Table 1:** Hydrogen-bond geometry (Å, °)

*D*—H⋯*A*	*D*—H	H⋯*A*	*D*⋯*A*	*D*—H⋯*A*
C1—H1*A*⋯O1^i^	0.93	2.45	3.378 (3)	172
C2—H2*B*⋯O3^ii^	0.93	2.46	3.356 (4)	162

Symmetry codes: (i) 
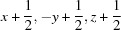
; (ii) 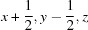.

**Fig. 1.**
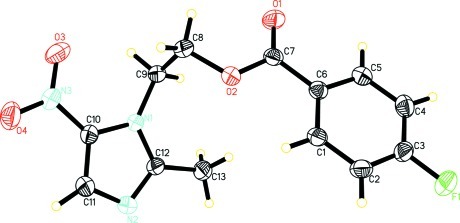
The molecular structure of the title compound with displacement ellipsoids drawn at 30% probability level.

**Fig. 2.**
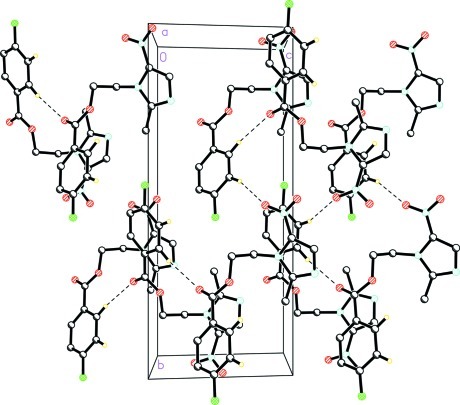
The crystal packing view of the title compound.

## supplementary crystallographic information

### Comment

The title compound is a derivative of well known broad spectrum antibiotic metronidazole, commonly known as Flagyl. A number of metronidazole derivatives have been synthesized to evaluate their biological potentials, such as antibacterial (Atia, 2009; Dubey *et al.*, 2009; Beena *et al.*, 2009; Bowden & Izadi, 1998), anticancer (Qian *et al.*, 2010), and *H. pylori* urease inhibitors (Mao *et al.*, 2009). These properties of metronidazole derivatives attracted the attention of synthetic and medicinal chemists to further explore their potential against different diseases. In present study, metronidazole ester derivative was prepared in a cost effective manner to evaluate its antiglycation potential.

In the title compound, the benzene (C1–C6) and imidazole (N1/C10/C11/N2/C12) rings are almost planar (Fig. 1) with a dihedral angle of 32.77 (12)° between the mean planes. The bond lengths and angles are within the normal ranges. C1—H1A···O1^i^ and C2—H2B···O3^ii^ hydrogen bonds (symmetry codes as in Table 1) play important roles in stabilizing the crystal structure by forming a three-dimensional network (Fig. 2).

### Experimental

The synthesis of title compounds 1 was achieved by reacting metronidazole (171 mg, 1.0 mmole) with 4-fluorobenzoic acid (1.2 equiv.) in the presence of dicyclohexylcarbodiimide (245 mg, 1.2 mmole) and 4-dimethylaminopyridine (0.35 mmole) in dichloromethane (10 ml) at room temperature for 40–45 h. The progress of reaction was monitored by TLC. The reaction was quenched with 20 ml HCl (0.5 *M*) and then basified with sat.NaHCO_3_. It was extracted with dichloromethane and evaporated in vacuo to obtain crude product. The mixture of crude product was purified by using silica gel chromatography (EtOAc:hexane, 3.0:7.0 to 7.0:3.0) which afforded compound 1 in 85% yield. Recrystallization from the slow evaporation of dichloromethane afforded pure crystals found suitable for single-crystal X-ray diffraction studies. All chemicals were purchased from Sigma–Aldrich.

### Refinement

H atoms on methyl, methylene and methine groups were positioned geometrically with C—H = 0.96, 0.97 and 0.93 Å, respectively, and constrained to ride on their parent atoms with *U*_iso_(H) = 1.2*U*_eq_(CH and CH_2_) and 1.5*U*_eq_(CH_3_). A rotating group model was applied to the methyl groups. In the absence of significant anomalous scattering effects, 952 Friedel pairs were merged before the final refinement.

### Crystal data

**Table:**

C_13_H_12_FN_3_O_4_	*F*(000) = 608
*M**_r_* = 293.26	*D*_x_ = 1.498 Mg m^−^^3^
Monoclinic, *C**c*	Mo *K*α radiation, λ = 0.71073 Å
*a* = 8.9669 (12) Å	Cell parameters from 2305 reflections
*b* = 18.784 (2) Å	θ = 2.6–28.0°
*c* = 7.8288 (10) Å	µ = 0.12 mm^−^^1^
β = 99.684 (3)°	*T* = 273 K
*V* = 1299.9 (3) Å^3^	Block, colourless
*Z* = 4	0.50 × 0.29 × 0.16 mm

### Data collection

**Table:**

Bruker SMART APEX CCD diffractometer	1186 independent reflections
Radiation source: fine-focus sealed tube	1150 reflections with *I* > 2σ(*I*)
Graphite monochromator	*R*_int_ = 0.022
ω scans	θ_max_ = 25.5°, θ_min_ = 2.2°
Absorption correction: multi-scan (*SADABS*; Bruker, 2000)	*h* = −10→10
*T*_min_ = 0.941, *T*_max_ = 0.981	*k* = −22→22
3782 measured reflections	*l* = −9→8

### Refinement

**Table:**

Refinement on *F*^2^	Secondary atom site location: difference Fourier map
Least-squares matrix: full	Hydrogen site location: inferred from neighbouring sites
*R*[*F*^2^ > 2σ(*F*^2^)] = 0.032	H-atom parameters constrained
*wR*(*F*^2^) = 0.088	*w* = 1/[σ^2^(*F*_o_^2^) + (0.0593*P*)^2^ + 0.2616*P*] where *P* = (*F*_o_^2^ + 2*F*_c_^2^)/3
*S* = 1.06	(Δ/σ)_max_ < 0.001
1186 reflections	Δρ_max_ = 0.15 e Å^−^^3^
192 parameters	Δρ_min_ = −0.11 e Å^−^^3^
2 restraints	Extinction correction: *SHELXL97* (Sheldrick, 2008), Fc^*^=kFc[1+0.001xFc^2^λ^3^/sin(2θ)]^-1/4^
Primary atom site location: structure-invariant direct methods	Extinction coefficient: 0.0055 (15)

### Special details

**Table:**

Geometry. All e.s.d.'s (except the e.s.d. in the dihedral angle between two l.s. planes) are estimated using the full covariance matrix. The cell e.s.d.'s are taken into account individually in the estimation of e.s.d.'s in distances, angles and torsion angles; correlations between e.s.d.'s in cell parameters are only used when they are defined by crystal symmetry. An approximate (isotropic) treatment of cell e.s.d.'s is used for estimating e.s.d.'s involving l.s. planes.
Refinement. Refinement of *F*^2^ against ALL reflections. The weighted *R*-factor *wR* and goodness of fit *S* are based on *F*^2^, conventional *R*-factors *R* are based on *F*, with *F* set to zero for negative *F*^2^. The threshold expression of *F*^2^ > σ(*F*^2^) is used only for calculating *R*-factors(gt) *etc*. and is not relevant to the choice of reflections for refinement. *R*-factors based on *F*^2^ are statistically about twice as large as those based on *F*, and *R*-factors based on ALL data will be even larger.

### Fractional atomic coordinates and isotropic or equivalent isotropic displacement parameters (Å^2^)

**Table:**

	*x*	*y*	*z*	*U*_iso_*/*U*_eq_	
O1	−0.1173 (2)	0.24543 (13)	−0.1523 (3)	0.0625 (6)	
O2	0.0800 (2)	0.27301 (10)	0.0543 (2)	0.0534 (5)	
O3	0.0473 (3)	0.48885 (12)	0.3047 (4)	0.0795 (8)	
O4	0.2371 (3)	0.50643 (12)	0.5096 (4)	0.0726 (7)	
N1	0.0456 (2)	0.34305 (11)	0.3929 (3)	0.0405 (5)	
N2	0.1729 (3)	0.29745 (13)	0.6362 (3)	0.0532 (6)	
N3	0.1392 (3)	0.46795 (12)	0.4283 (3)	0.0516 (6)	
C1	0.1985 (3)	0.13945 (15)	0.0541 (3)	0.0454 (6)	
H1A	0.2496	0.1741	0.1262	0.054*	
C2	0.2552 (3)	0.07076 (17)	0.0578 (4)	0.0539 (7)	
H2B	0.3442	0.0587	0.1312	0.065*	
C3	0.1764 (3)	0.02112 (15)	−0.0496 (4)	0.0533 (7)	
C4	0.0462 (3)	0.03555 (17)	−0.1617 (4)	0.0554 (7)	
H4A	−0.0035	0.0004	−0.2335	0.066*	
C5	−0.0093 (3)	0.10470 (15)	−0.1646 (4)	0.0481 (6)	
H5A	−0.0978	0.1163	−0.2397	0.058*	
C6	0.0659 (3)	0.15675 (14)	−0.0565 (3)	0.0408 (6)	
C7	−0.0021 (3)	0.22884 (14)	−0.0609 (3)	0.0429 (6)	
C8	0.0198 (4)	0.34362 (15)	0.0713 (4)	0.0569 (7)	
H8A	−0.0514	0.3554	−0.0323	0.068*	
H8B	0.1013	0.3781	0.0840	0.068*	
C9	−0.0587 (3)	0.34704 (14)	0.2269 (4)	0.0474 (6)	
H9A	−0.1153	0.3912	0.2230	0.057*	
H9B	−0.1304	0.3081	0.2208	0.057*	
C10	0.1340 (3)	0.39608 (14)	0.4807 (4)	0.0454 (6)	
C11	0.2095 (3)	0.36676 (16)	0.6288 (4)	0.0535 (7)	
H11A	0.2764	0.3909	0.7126	0.064*	
C12	0.0761 (3)	0.28463 (14)	0.4928 (4)	0.0435 (6)	
C13	0.0081 (4)	0.21350 (15)	0.4469 (4)	0.0579 (8)	
H13A	0.0472	0.1798	0.5353	0.087*	
H13B	0.0332	0.1984	0.3381	0.087*	
H13C	−0.0998	0.2165	0.4376	0.087*	
F1	0.2301 (3)	−0.04671 (11)	−0.0425 (3)	0.0827 (7)	

### Atomic displacement parameters (Å^2^)

**Table:**

	*U*^11^	*U*^22^	*U*^33^	*U*^12^	*U*^13^	*U*^23^
O1	0.0549 (11)	0.0618 (12)	0.0642 (14)	0.0139 (10)	−0.0092 (10)	0.0027 (11)
O2	0.0660 (12)	0.0480 (11)	0.0422 (11)	0.0104 (9)	−0.0022 (9)	−0.0033 (8)
O3	0.0938 (17)	0.0483 (12)	0.0877 (18)	0.0064 (12)	−0.0103 (15)	0.0188 (12)
O4	0.0740 (14)	0.0472 (11)	0.0926 (18)	−0.0096 (11)	0.0026 (13)	−0.0116 (12)
N1	0.0454 (10)	0.0358 (10)	0.0395 (12)	0.0050 (8)	0.0048 (9)	−0.0012 (9)
N2	0.0660 (14)	0.0472 (12)	0.0429 (14)	0.0034 (11)	−0.0012 (11)	0.0026 (10)
N3	0.0562 (12)	0.0392 (11)	0.0598 (15)	0.0043 (10)	0.0113 (11)	−0.0010 (11)
C1	0.0473 (13)	0.0508 (14)	0.0371 (14)	0.0048 (11)	0.0039 (11)	0.0013 (11)
C2	0.0525 (15)	0.0622 (18)	0.0451 (15)	0.0134 (13)	0.0028 (12)	0.0075 (13)
C3	0.0732 (19)	0.0435 (15)	0.0463 (17)	0.0125 (13)	0.0193 (14)	0.0061 (12)
C4	0.0673 (17)	0.0485 (14)	0.0510 (17)	−0.0084 (13)	0.0119 (14)	−0.0089 (13)
C5	0.0475 (13)	0.0529 (15)	0.0424 (15)	−0.0014 (11)	0.0033 (11)	−0.0002 (11)
C6	0.0417 (11)	0.0455 (13)	0.0359 (14)	0.0014 (10)	0.0088 (10)	0.0009 (10)
C7	0.0454 (13)	0.0484 (15)	0.0347 (14)	0.0049 (10)	0.0064 (10)	0.0034 (11)
C8	0.085 (2)	0.0412 (15)	0.0427 (16)	0.0099 (13)	0.0049 (14)	0.0064 (11)
C9	0.0532 (14)	0.0419 (13)	0.0427 (15)	0.0073 (11)	−0.0049 (11)	−0.0005 (11)
C10	0.0480 (12)	0.0395 (13)	0.0474 (16)	0.0047 (10)	0.0043 (11)	−0.0050 (10)
C11	0.0553 (14)	0.0476 (14)	0.0531 (17)	0.0011 (12)	−0.0038 (12)	−0.0071 (13)
C12	0.0517 (13)	0.0381 (12)	0.0406 (15)	0.0044 (11)	0.0079 (11)	0.0033 (10)
C13	0.082 (2)	0.0397 (14)	0.0501 (18)	−0.0051 (14)	0.0053 (15)	0.0026 (13)
F1	0.1168 (17)	0.0497 (11)	0.0819 (15)	0.0242 (11)	0.0175 (13)	0.0035 (10)

### Geometric parameters (Å, º)

**Table:**

O1—C7	1.195 (3)	C3—C4	1.364 (4)
O2—C7	1.349 (3)	C4—C5	1.390 (4)
O2—C8	1.447 (3)	C4—H4A	0.9300
O3—N3	1.225 (3)	C5—C6	1.391 (4)
O4—N3	1.229 (4)	C5—H5A	0.9300
N1—C12	1.349 (3)	C6—C7	1.483 (3)
N1—C10	1.382 (3)	C8—C9	1.507 (4)
N1—C9	1.470 (4)	C8—H8A	0.9700
N2—C12	1.321 (4)	C8—H8B	0.9700
N2—C11	1.346 (4)	C9—H9A	0.9700
N3—C10	1.414 (4)	C9—H9B	0.9700
C1—C2	1.385 (4)	C10—C11	1.357 (4)
C1—C6	1.387 (3)	C11—H11A	0.9300
C1—H1A	0.9300	C12—C13	1.487 (4)
C2—C3	1.370 (4)	C13—H13A	0.9600
C2—H2B	0.9300	C13—H13B	0.9600
C3—F1	1.360 (3)	C13—H13C	0.9600
			
C7—O2—C8	117.1 (2)	O2—C7—C6	111.7 (2)
C12—N1—C10	104.7 (2)	O2—C8—C9	110.2 (2)
C12—N1—C9	126.2 (2)	O2—C8—H8A	109.6
C10—N1—C9	129.0 (2)	C9—C8—H8A	109.6
C12—N2—C11	105.6 (2)	O2—C8—H8B	109.6
O3—N3—O4	123.2 (3)	C9—C8—H8B	109.6
O3—N3—C10	119.0 (2)	H8A—C8—H8B	108.1
O4—N3—C10	117.8 (3)	N1—C9—C8	113.4 (2)
C2—C1—C6	120.3 (3)	N1—C9—H9A	108.9
C2—C1—H1A	119.9	C8—C9—H9A	108.9
C6—C1—H1A	119.9	N1—C9—H9B	108.9
C3—C2—C1	118.2 (3)	C8—C9—H9B	108.9
C3—C2—H2B	120.9	H9A—C9—H9B	107.7
C1—C2—H2B	120.9	C11—C10—N1	107.1 (2)
F1—C3—C4	118.1 (3)	C11—C10—N3	126.8 (3)
F1—C3—C2	118.1 (3)	N1—C10—N3	126.0 (2)
C4—C3—C2	123.8 (3)	N2—C11—C10	109.9 (3)
C3—C4—C5	117.5 (3)	N2—C11—H11A	125.1
C3—C4—H4A	121.3	C10—C11—H11A	125.1
C5—C4—H4A	121.3	N2—C12—N1	112.6 (2)
C4—C5—C6	120.7 (3)	N2—C12—C13	123.6 (2)
C4—C5—H5A	119.6	N1—C12—C13	123.8 (3)
C6—C5—H5A	119.6	C12—C13—H13A	109.5
C1—C6—C5	119.5 (2)	C12—C13—H13B	109.5
C1—C6—C7	122.3 (2)	H13A—C13—H13B	109.5
C5—C6—C7	118.2 (2)	C12—C13—H13C	109.5
O1—C7—O2	124.0 (2)	H13A—C13—H13C	109.5
O1—C7—C6	124.2 (3)	H13B—C13—H13C	109.5
			
C6—C1—C2—C3	−0.3 (4)	O2—C8—C9—N1	70.0 (3)
C1—C2—C3—F1	−178.3 (2)	C12—N1—C10—C11	−1.2 (3)
C1—C2—C3—C4	0.8 (4)	C9—N1—C10—C11	179.3 (3)
F1—C3—C4—C5	178.4 (3)	C12—N1—C10—N3	−178.8 (3)
C2—C3—C4—C5	−0.6 (4)	C9—N1—C10—N3	1.7 (4)
C3—C4—C5—C6	−0.1 (4)	O3—N3—C10—C11	−168.7 (3)
C2—C1—C6—C5	−0.4 (4)	O4—N3—C10—C11	11.5 (4)
C2—C1—C6—C7	177.9 (2)	O3—N3—C10—N1	8.4 (4)
C4—C5—C6—C1	0.6 (4)	O4—N3—C10—N1	−171.3 (3)
C4—C5—C6—C7	−177.8 (2)	C12—N2—C11—C10	0.3 (3)
C8—O2—C7—O1	2.6 (4)	N1—C10—C11—N2	0.6 (3)
C8—O2—C7—C6	−175.7 (2)	N3—C10—C11—N2	178.2 (3)
C1—C6—C7—O1	−178.7 (3)	C11—N2—C12—N1	−1.1 (3)
C5—C6—C7—O1	−0.4 (4)	C11—N2—C12—C13	179.0 (3)
C1—C6—C7—O2	−0.4 (3)	C10—N1—C12—N2	1.4 (3)
C5—C6—C7—O2	177.9 (2)	C9—N1—C12—N2	−179.0 (2)
C7—O2—C8—C9	99.1 (3)	C10—N1—C12—C13	−178.6 (2)
C12—N1—C9—C8	−99.5 (3)	C9—N1—C12—C13	0.9 (4)
C10—N1—C9—C8	79.9 (3)		

### Hydrogen-bond geometry (Å, º)

**Table:**

*D*—H···*A*	*D*—H	H···*A*	*D*···*A*	*D*—H···*A*
C1—H1*A*···O1^i^	0.93	2.45	3.378 (3)	172
C2—H2*B*···O3^ii^	0.93	2.46	3.356 (4)	162

Symmetry codes: (i) *x*+1/2, −*y*+1/2, *z*+1/2; (ii) *x*+1/2, *y*−1/2, *z*.
